# Modification of glass-ionomer cement properties by quaternized chitosan-coated nanoparticles

**DOI:** 10.1007/s10266-022-00738-0

**Published:** 2022-09-07

**Authors:** Enas A. Elshenawy, Manal Ahmed El-Ebiary, El-Refaie Kenawy, Gehan Abdelmonem El-Olimy

**Affiliations:** 1 Dental Biomaterials Department, Faculty of Dentistry, Tanta University, Tanta, 31773 Egypt; 2 Chemistry Department, Faculty of Science, Tanta University, Tanta, 31111 Egypt

**Keywords:** Glass-ionomer cements, Mechanical properties, Wear resistance, Antibacterial activity, Fluoride release

## Abstract

Glass ionomers (GICs), because of their qualities, are in a good position to be modified to resist masticatory stresses as permanent posterior restoration and prevent recurrent caries. The purpose of the present study was to evaluate the effect of adding quaternized chitosan-coated mesoporous silica nanoparticles (HTCC@MSNs) to conventional GIC on its mechanical properties, antimicrobial activity and fluoride release and the effect of 1- and 3-month water aging on the studied properties. HTCC@MSNs was synthesized, added to commercially available conventional GIC at 1%, 3%, and 5% by weight forming three experimental groups and compared with plain GIC as a control group. Flexural strength, modulus, Vickers microhardness and wear volumes were evaluated. Antibacterial activity was tested against *Streptococcus mutans* and fluoride release in de-ionized water was measured. All properties were evaluated before and after one- and three-month aging (*n* = 10 specimens per test/per time). Two-way ANOVA was used for statistical analysis. Characterization confirmed successful preparation of HTCC@MSNs. The flexural strength, modulus, hardness and wear resistance of the GICs improved significantly by adding 1–3% HTCC@MSNs, while 5% HTCC@MSNs group showed no significant difference compared to control group. Bacterial inhibition zones and fluoride release increased proportionally to the amount of filler added. Mechanical properties were improved by artificial aging. Fluoride release values, and bacterial inhibition zones decreased with aging for all groups. HTCC@MSNs as a filler with the optimized proportion provides strengthening and antibacterial effect. In addition, aging is an important factor to be considered in evaluating experimental fillers.

## Introduction

Glass-ionomer cements (GICs) are widely applied in dentistry for a variety of purposes. GICs have unique qualities such as bulk insertion, chemical bond to the dental hard tissues, fluoride ion release and recharge, biocompatibility, thermal expansion coefficient comparable to enamel and dentin, and satisfactory esthetics. Even with these features, the properties of GIC may not be enough to overcome the constraints in areas of heavier occlusion. High viscosity GICs, with the size of fine glass particle and greater cross-linking, showed improved mechanical properties and lower solubility [1]. Nevertheless, lack of strength remains a serious problem for GICs in dental applications [2].

In addition, biofilms grow on dental and GIC surfaces because of the broad variety of bacteria species in the oral cavity, as well as the complexity of the GIC’s surface chemistry and rough surface [3]. Thus, a modification that achieves both strengthening and antibacterial effect is a necessity for a less technique sensitive material as GIC. The controlled release of active ingredients from the GIC matrix through a mesoporous filler may achieve the required purpose of enhancing the mechanical properties and augmenting fluoride in preventing recurrent caries.

Mesoporous silica nanoparticles (MSNs) have piqued a considerable research interest due to their highly favorable morphological features such as extensive specific surface area, porous structure, convenient surface modification, biocompatibility as well as appealing physicochemical properties with the ability to act as a drug delivery system [4]. MSNs are chemically stable, resistant to microbial attack, and have greater mechanical strength since they are inorganic nanomaterials [5]. In a study by Zhang et al. [6], chlorhexidine-encapsulated MSNs (CHX@MSN) were fabricated and added to the resin composite after silanization. They found that the composites with CHX@MSN showed a controlled release of CHX and retained mechanical properties to a great extent, while composites with only CHX showed abrupt release of CHX and a decline in mechanical properties.

Other studies have shown that addition of mesoporous silica nanoparticles alone or in combination with nonporous filler into resin composite improved the mechanical qualities [7, 8]. This improvement was explained by the increased surface area of the nanosized filler and the porous structure that helps in more efficient bond between the filler and the matrix. Smaller particles, on the other hand, allow more particles to share the stresses applied [9].

The antimicrobial agents commonly used for GICs modification were antiseptics [10], biomaterials [11, 12], antibiotics [13]in addition to some natural products [14, 15]. However, limited data on the effect of these additives on the mechanical properties have been reported. Chitosan is a natural polymer generated from the partial deacetylation of chitin, which can be found in crab shells. Chitosan is used in a wide range of biomedical applications due to its biocompatibility, biodegradability, hydrophilicity, hemostatic, and anti-antigenic characteristics. The incorporation of chitosan in glass ionomer cements was reported to not only enhance the antibacterial activity, but also to improve flexural and compressive strength of the cement [16]. Chitosan variants with quaternary ammonium groups have been proven to have stronger antibacterial activity than the parent chitosan [17]. This was due to the higher positive charge that can readily lead to more bacterial cell destruction and thus stronger antibacterial action [18]. As one of quaternized chitosans, N-[(2-hydroxy-3-trimethylammonium) propyl] chitosan (HTCC) has good antibacterial effect against *S. aureus, E. coli*, *C. albicans* [19] and *S. mutans* [20].

Longevity is regarded as one of the most important considerations of filling materials by both dentists and patients. As a result, dental restorative materials must be evaluated to see if they are prone to degradation over time [21]. Water aging is a frequently used experimental approach for evaluating the performance of materials and simulating the physiological aging of biomaterials [22].

To our knowledge, the utilization of quaternized chitosan-coated mesoporous silica nanoparticles (HTCC@MSNs) as a filler to modify GICs has not been studied before. This study is designed to assess the hypothesis that adding HTCC@MSNs to GIC and aging will have a strengthening effect on its physico-mechanical properties and enhance its antibacterial capability and fluoride release ability. In the present study different ratios of HTCC@MSNs were added as a filler to GIC and their effect on physico-mechanical properties, fluoride release and antibacterial qualities of GICs was evaluated, in addition the effect of artificial aging for 1 and 3 months was studied.

## Materials and methods

The details of the used materials in preparation of the filler, control and experimental specimens including their composition details and batch numbers are listed in Table 1.

**Table 1:** Materials used in the study

Study stage	Material	Identification
Preparation of mesoporous silica	Tetraethyl orthosilicate (TEOS)^a^ Sodium hydroxide^b^ Cetyltrimethylammonium bromide (CTAB)^b^ Ethanol^b^	Batch number:26.1740703 CAS number: 1310-73-2 CAS:57-09-0 CAS number: 64-17-5
Preparation of quaternized chitosan	Chitosan^**b**^ (Low molecular weight) Glycidyltrimethylammonium chloride (GTMAC)^b^ Acetone^**b**^ Perchloric acid^**c**^	CAS number:9012-76-4, catalog number: 448869 CAS number: 3033-77-0 CAS number: 67-64-1 CAS number: 7601-90-3
Reagents for functionalization	Methanol^**b**^	CAS number: 67-56-1
Preparation of GIC samples	Fuji IX GP glass ionomer (MINIPACK)^**d**^ Composition: polyacrylic acid, Fluoroaluminosilicate glass, other ingredients	Batch number: 002578

^a^chem-lab, Zedelgem, Belgium

^b^Sigma-Aldrich, MO, USA

^c^Dop Organik Kimya, Istanbul, Turkey

^d^GC corporation, Tokyo, Japan

### Synthesis and characterization of the filler

MSNs were prepared as reported by Cao et al. [23]. The architecture was directed by CTAB and the silica supply was TEOS. CTAB (1 g) was dispersed in 480 mL distilled water, 3.5 mL NaOH (2 M) was added under continual stirring for 20 min. In an oil bath, this solution was heated at 80 °C, then TEOS (5 mL) was introduced drop by drop (1 mL/min) and vigorously stirred for 2 h. The resultant white precipitate was washed using ethanol/distilled water. To entirely eliminate surfactant, resultant product was heat treated at 550 °C for 5 h.

Quaternization of chitosan was done according to Ruihua et al. [24]**.** At room temperature, chitosan (2 g) was dispersed in 30 mL distilled water then entirely dissolved in 1.14 mL perchloric acid. To enable pre-reaction with chitosan, GTMAC (13.3 mL) was added in three fractional parts to chitosan suspension at 60 °C at 30 min break, followed by 8-h reaction at 80 °C. The mixture was precipitated by pouring it into acetone, then filtered and dried in a 60 °C oven. Functionalization was performed as Cao et al. [23]**.** The prepared MSNs (60 mg) were dispersed in 1 mL methanol. The suspension was subjected to 30-min low-power sonication. After that, 2.4 mL of an aqueous solution of HTCC (25 mg/mL) was dropped into the suspension and further sonification continued for 10 min. HTCC@MSNs were recovered via 10 min-centrifuge (10,000 rpm), 3 times wash using distilled water and lyophilized.

### Morphologic and chemical characterization

Shape and particle size of produced particles were investigated via scanning electron microscope “SEM” (JEOL-JSM-5200LV, Tokyo, Japan). Gold was sputter deposited onto the samples. Then, magnifications ranging from 7500 to 27,000 times were used to inspect the samples. Transmission electron microscope “TEM” (JEM-2100F, JEOL, Japan) was employed in revealing the structural features of mesoporous silica particles before and after functionalization. Fourier-transform infrared spectroscopy **“**FTIR” was used to pinpoint functional group on prepared MSNs particles, HTCC and HTCC@MSNs. Each sample powder was pressed into a pellet with potassium bromide (KBr). Tensor 27 Bruker spectrometer (Bruker Optik GmbH, Germany) was used to record IR spectra in the 4000–400 cm^−1^ range. The obtained FTIR spectra were assessed and analyzed for the typical SiO_2_ spectra, presence of quaternary NH_2_ groups and to validate surface-coating by HTCC onto the MSNs.

The crystalline nature of the samples was analyzed using X-ray diffraction “XRD” Diffractometer (GNR APD-2000 PRO, Toryno, Italy). It used a spinning x-ray generator with 40 kW and 40 mA. Nanoparticle XRD data were captured from 5° to 80° using a 0.02 θ step size and a 0.3°/min scanning speed.

The zeta potential of the nanoparticles was examined utilizing Malvern ZetaSizer Nano ZS Analyzer. Water has been used as a dispersant to make suspensions with concentrations of 1 mg/mL.

### Filler incorporation into glass ionomer cement and specimen preparation

A balance with an accuracy of 0.0001 g was used to weigh the filler powders. For the preparation of experimental groups,1%,3%, and 5% by weight of HTCC@MSNs were added to the powder using the geometric dilution method before mixing with the liquid.

Thirty specimens per test were prepared for each group. Ten specimens were measured at each time interval. All specimens were prepared using the indicated proportion of 3.6 powder/1 liquid Fuji IX GP at room temperature. Using plastic spatula and polytetrafluoroethylene (PTFE) mold prepared with the specified dimensions for each test, specimens were 25–30 s hand mixed. Excess material was extruded by pressing glass microscope slides with transparent polystyrene matrix films against both sides of the specimen under manual pressure. After 1 h, specimens were extracted from their mold, grinded using wet silicon carbide (600-grit) and then they were kept in distilled water 24 h prior mechanical testing.

### Artificial aging procedure

Distilled water was used to store two divisions of samples from the control group and each experimental group. For one and three months, samples were incubated at 37 °C. Each group of ten specimens of the same material was immersed in 100 mL of water in a sealed, labeled glass bottle. To avoid saturation by degradation product, the water was changed once a week [25].

### Properties evaluation

The tested groups were: control group (0% HTCC@MSNs), 1% HTCC@MSNs, 3% HTCC@MSNs, and 5% HTCC@MSNs. For all groups, thirty specimens were made for each property. Ten specimens were evaluated at specified time; after specimen preparation, after 1-month aging and after 3-month aging.

Sample size was calculated using a computer program G power version 3. The minimum number for sample size was 10 specimens in each group. The sample was collected based on previous study [7]. The significance level was 0.05 and the power sample size was more than 80% or this study and the confidence interval 95%.

#### Flexural strength test and elastic modulus evaluation

Flexural strength specimens of 2 mm thickness, 2 mm width and 25 mm length were prepared. Specimens were subjected to three-point bending measurement using a Universal Testing Machine (Instron 3365 series, USA). The test assembly was made up of two -20 mm- apart supporting wedges and a loading wedge that applied load at 1.0 mm/min crosshead speed until specimen fracture. Using Bluhill software (Bluehill 3 Testing Software, USA), the maximum load was recorded and flexural strength (MPa) was determined.

Elastic modulus (E) (GPa) was calculated after recording flexural strength by the following equation [26]: E = FL^3^/4BH^3^d, where F, B, L, D and H were applied load, specimen width, support span, deflection (mm), and specimen thickness, respectively.

#### Vickers microhardness

Cylindrical specimens of 3 mm height and 6 mm diameter were prepared. Specimen surfaces were finished by hand-grinding using wet 800-,1000-, and 1200-grit silicon carbide paper after setting. Vickers hardness measurements were taken using a Digital Microhardness Tester (Zwick/Roell, INDENTEC, ZHVµ-S, West Midlands, England). With a dwell time of 30 s, a 50 gf load was delivered through the indenter. Each specimen had three indentations made on its surface. The diagonal lengths of the indentations were viewed through 10X objective lens (Clemex S 1.3 M # 1,553,105; Clemex Technologies Inc) and measured with a built-in scaled microscope. The hardness number (VHN) was calculated using the machine’s software.

#### Wear resistance assessment

Wear resistance was measured using standardized dynamic loading method by chewing simulator. (Chewing simulator CS-4.4, SD Mechatronik GMBH, Germany).

Ten disc-shaped specimens (12 mm of diameter and 3.0 mm of thickness) for each group were prepared using a Teflon mold. The GIC samples were removed from water and the top surfaces were wet-ground with an 800- and 1200-grit silicon carbide paper.

The samples were resin mounted and their outer surfaces were marked with orientation grooves to make digital matching of each sample and calculate the difference before and after the chewing simulation. The process of chewing simulation was achieved by two moving parts: the vertical bar (holding a 4 mm steel ball antagonist with the ability to move up and down) and the horizontal table (where the mounted specimens performed both protrusive and retrusive motions, controlled by a servo motor). The load was set to 40 N, to simulate the average clinical chewing forces in the molar region. In this process, the indenter was moved with an average downward speed of 40 mm/s to the test surface, while sliding for a distance of 2 mm with a lateral speed of 20 mm/s, and finally moved upwards with a speed of 60 mm/s. Specimens were subject to 30,000 wear cycles at a frequency of 0.96 Hz [27].

During the wear test, rinsing with distilled water was used to remove abraded particles from the sample surface [28].

The samples were optically scanned before and after being subjected to chewing simulation (baseline and follow up scans) using 3D scanner (DOF—Freedom HD Dental Scanner). The two scans of each specimen were imported independently into exocad software (exocad^®^ DentalCAD, version 2.2 Valletta, Exocad GmbH, Darmstadt, Germany) for analysis. Then, the exocad software allowed for an accurate superimposition of both (baseline and follow up) 3D surface scans of each specimen. Substance loss was indicated by the color mapping tool and volume measurement of the wear facets was made using mimics medical software (Mimics Innovation Suite 20, Materialise, Leuven, Belgium) and recorded in cubic millimeters.

#### Antibacterial activities

The antibacterial efficacy of the set specimens against *Streptococcus mutans* (ATCC 25,175) was investigated in vitro using standard disc diffusion method on brain heart infusion (BHI) agar plates. Cement discs (6 mm diameter, 3 mm thickness) were prepared with sterile instruments and sterilized by UV radiation (CAMAG UV Cabinet, CAMAG Germany) at 254 nm for 60 min before proceeding into the next step [29]. Mueller–Hinton agar plates (Sigma Aldrich, MO, USA) seeded with 1.8 × 10^8^ cfu/mL (0.5 OD^600^) of the test bacteria were checked for the presence of inhibition zones after 24 h of incubation at 37 °C. Inhibition zones surrounding the specimens were measured (mm) using a digital caliper at the outer limit of the inhibition zone generated considering only halos > 6 mm.

#### Fluoride release

Specimens with a diameter of 5 mm and a thickness of 3 mm were prepared and were immersed separately in 50 mL de-ionized water in closed glass bottle at 37 °C for 24 h after setting. The fluoride ion concentration was measured by removing the specimen and collecting the storage solution for examination. The 24 h-release of fluoride was measured at days 1, 7 and 14. Specimens were delicately dried before being placed in a new bottle with fresh 50 ml of de-ionized water. Specimens were transferred from the former bottle to the next every 24hrs from the first to the 14th day. The water in each bottle was collected on days 1, 7 and 14 for the measurement of fluoride release. 5 mL of total ionic strength adjustment buffer (TISAB III) solution was added to the collected solution to prevent formation of fluoride complexes. The ion-selective electrode method [30] was applied for measuring the fluoride ion concentration (μg/ml). The Total fluoride in μg was calculated by multiplying the values in μg/ml by the tested solution volume (50 ml). The total fluoride was then divided by the area of the specimen to obtain the fluoride release in μg/cm^2^.

### Statistical analysis

IBM SPSS software was used to collect data and perform statistical analysis. Shapiro–Wilk test was applied to test the normal distribution of the outcomes. Being normally distributed outcomes, two-way analysis of variance (ANOVA) was used to test the effect of different filler concentration and the effect of water aging on the evaluated properties. If needed, Tukey's test was performed for pair-wise comparisons. *P* ≤ 0.05 is defined as the significance level.

## Results

### Morphologic and chemical characterization

**Fig. 1:**
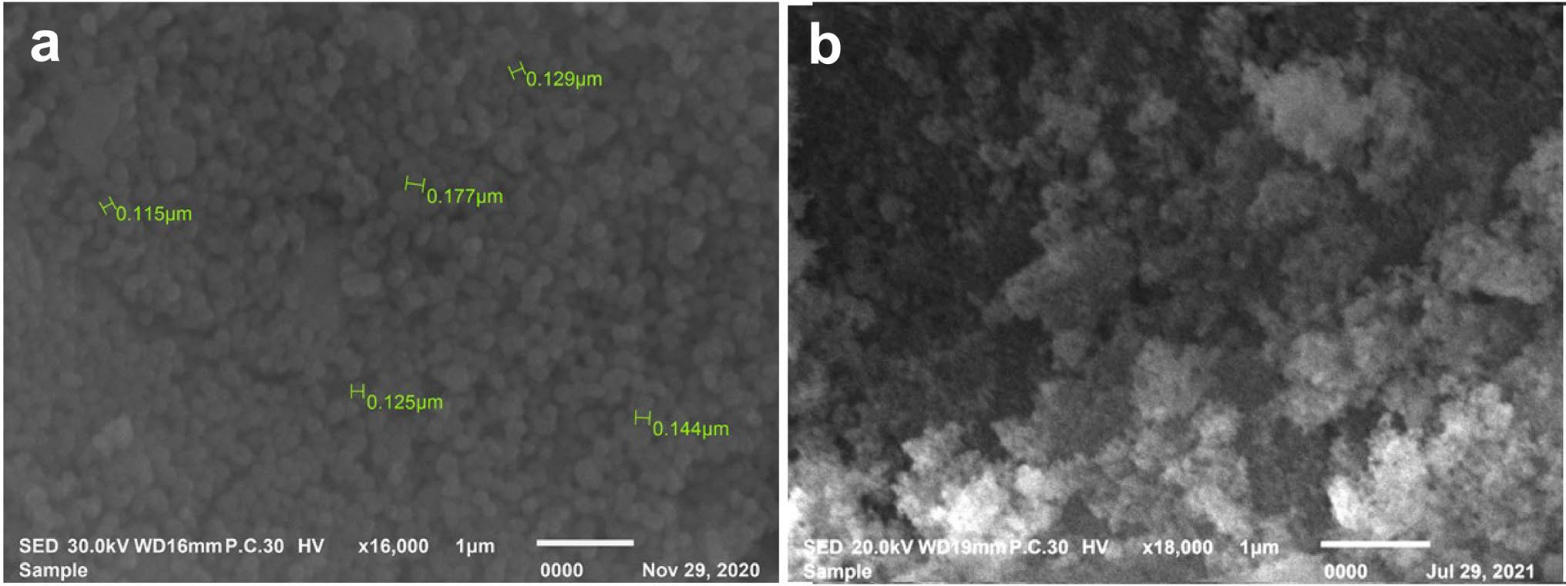
SEM micrographs of **a** MSNs showing smooth, uniform spherical shape and **b** HTCC@MSNs showing roughness after coating

**Fig. 2:**
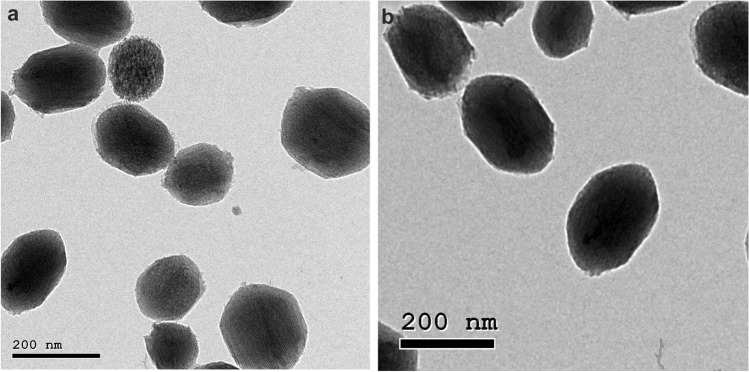
TEM images of **a** MSNs showing mesoporous texture, and **b** HTCC@MSNs showing obstruction of mesoporous texture

According to SEM micrographs Fig. 1, MSNs produced were nearly uniform spherical nanoparticles with reasonably smooth textures and particle diameters ranging from 115 to 177 nm. The MSNs' surfaces were clearly rough following HTCC coating. From Fig. 2(a), MSNs revealed a well-organized mesoporous network with a hexagonal arrangement, which is a structural feature. The absence of mesoporous surface texture is evident in Fig. 2(b). Morphological characterization by SEM showing roughness of the particles and TEM showing obscured mesoporous texture indicates that HTCC coating was achieved.

**Fig. 3:**
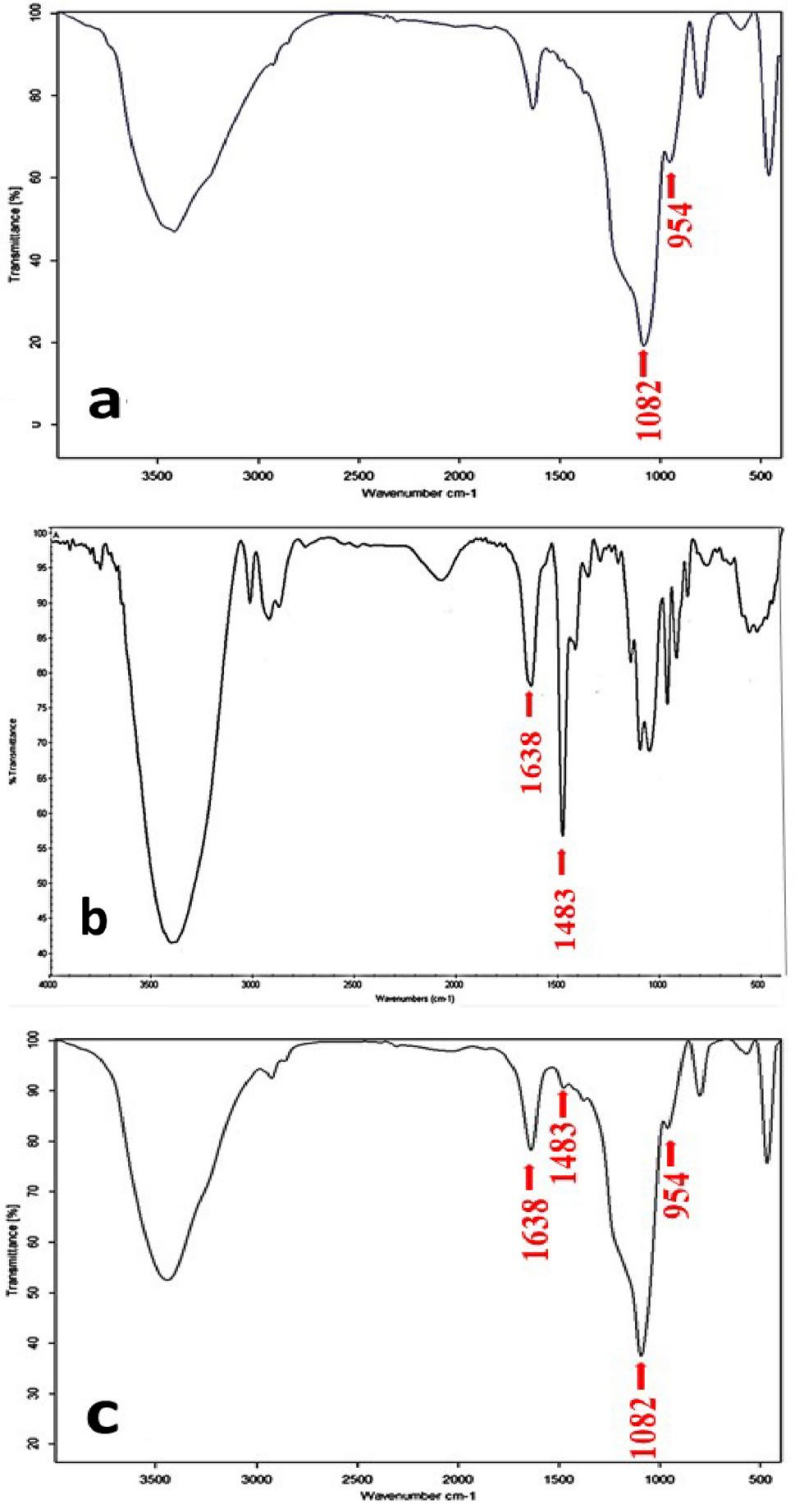
FTIR spectroscopy of **a** MSNs reflecting siloxane Si–O–Si stretching mode and Si–OH bending, **b** HTCC reflecting C–H and N–H bending mode, and **c** HTCC@MSNs

The FTIR spectroscopy of MSNs, HTCC, and HTCC@MSNs can be seen in Fig. 3. In the MSN samples, the wide absorption peak at 1082 cm^−1^ reflects siloxane (Si–O–Si) stretching mode. Si–OH bending was found to be responsible for the 954 cm^−1^ absorption band. No 2919,2890, and 1484 cm^−1^bands were found. Bending of C–H in –N(CH_3_)_3_^+^ is responsible for 1483 cm^−1^ peak in the HTCC spectra, the 1638 cm^−1^ peak reflects N–H bending mode. The typical absorption band of MSNs and HTCC are shown in HTTC@MSNs spectra. In the MSN samples, absence of 2919,2890, and 1484 cm^−1^ bands implies that there is no CTAB remnant [31]. Bending of C–H in –N(CH_3_)_3_^+^ in the HTCC spectra indicates quaternization of NH_2_ group in the chitosan structure [23]. The N–H bending mode found in HTCC spectra indicates existence of secondary amines due to the reactions at –NH_2_ sites on the chitosan structure [32].

**Fig. 4:**
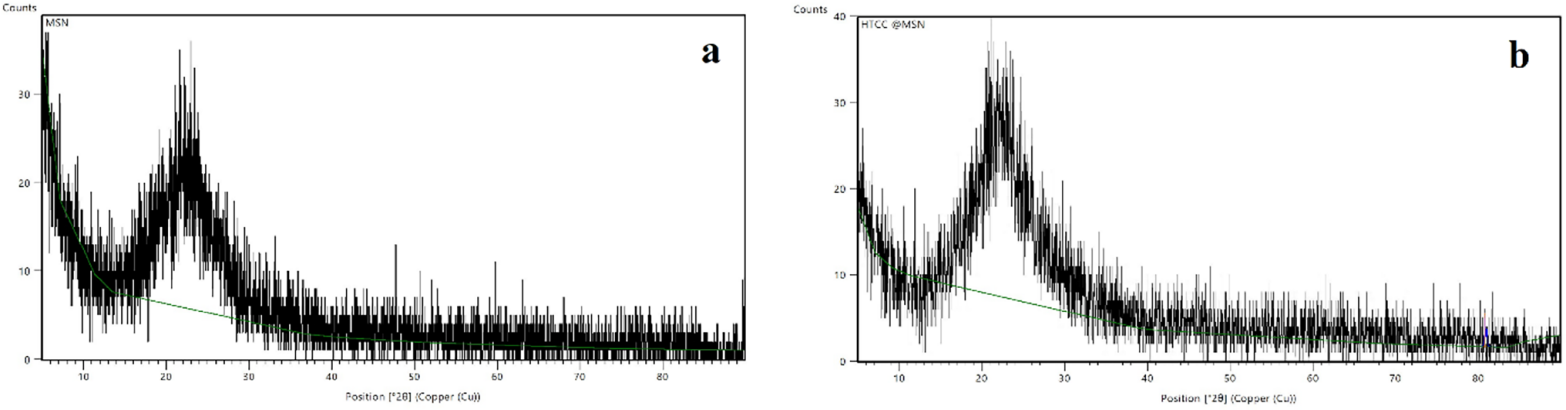
XRD patterns of **a** MSNs and **b** HTCC@MSNs showing the characteristic peak at 2 = 22–24 *θ* indicating amorphous structure

Characteristic diffractograms are depicted in Fig. 4. Both the HTCC-coated and the uncoated MSNs have amorphous structures. The remarkable diffraction pattern of silica amorphous phase was highlighted by the increased peak at roughly 2 = 22–24 *θ*. The porous structure before and after functionalization was confirmed by the remarkable diffraction angle of MSNs which related to presence of large pores with frequent periodic variations in the electronic density [33].

**Fig. 5:**
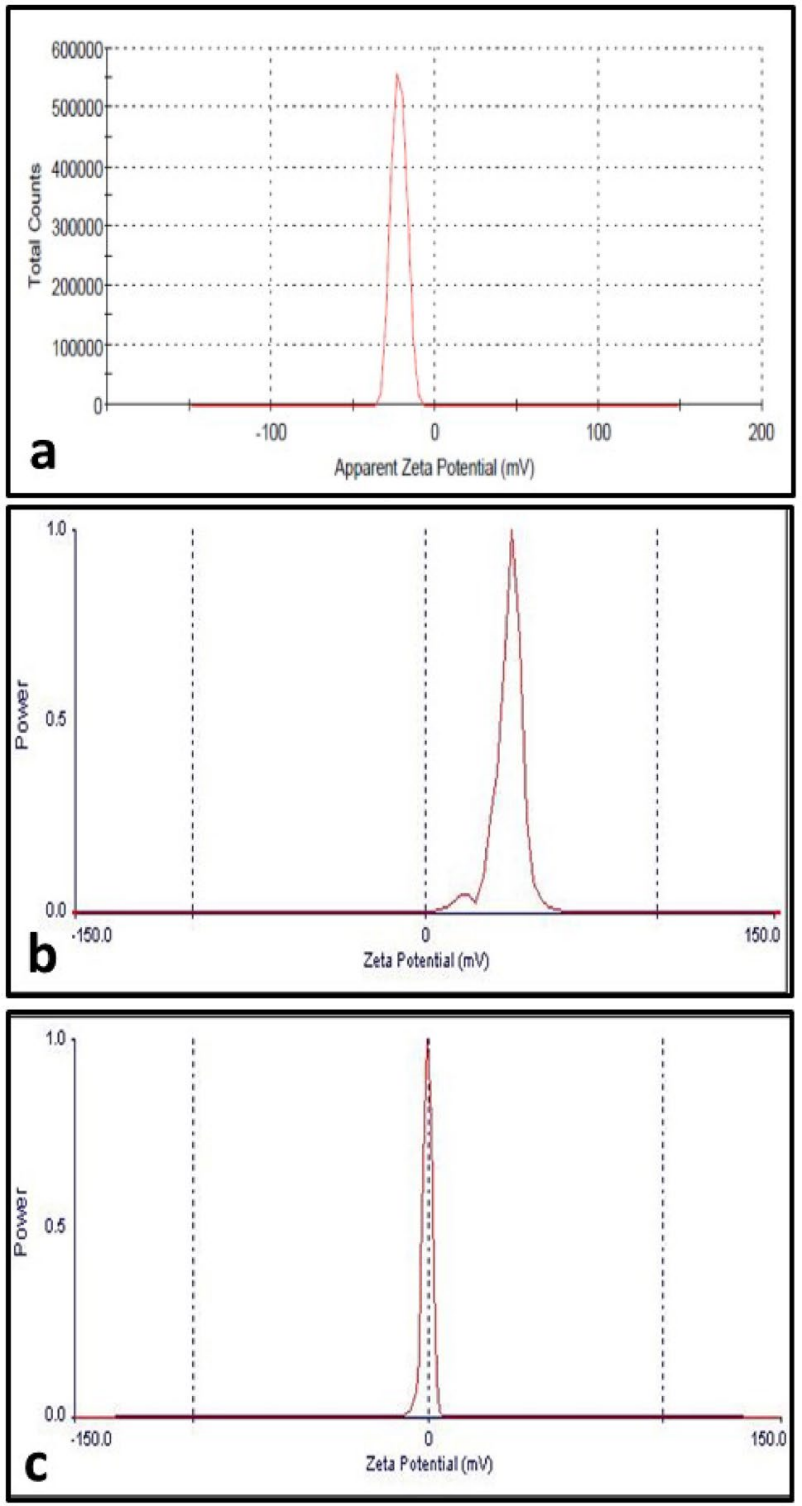
Zeta potential graphs of **a** MSNs **b** HTCC and **c** HTCC@MSNs

Zetapotential graphs (Fig. 5) show that bare MSNs have a negative charge (− 22.4 mV), HTCC has a positive charge (34.84 mV), and HTCC@MSNs has a positive charge (0.34 mV). The increase in zetapotential after coating from (− 22.4 mV) to (0.34 mV) indicates successful functionalization with HTCC [23].

### Properties evaluation

#### Flexural strength and flexural modulus values

**Fig. 6:**
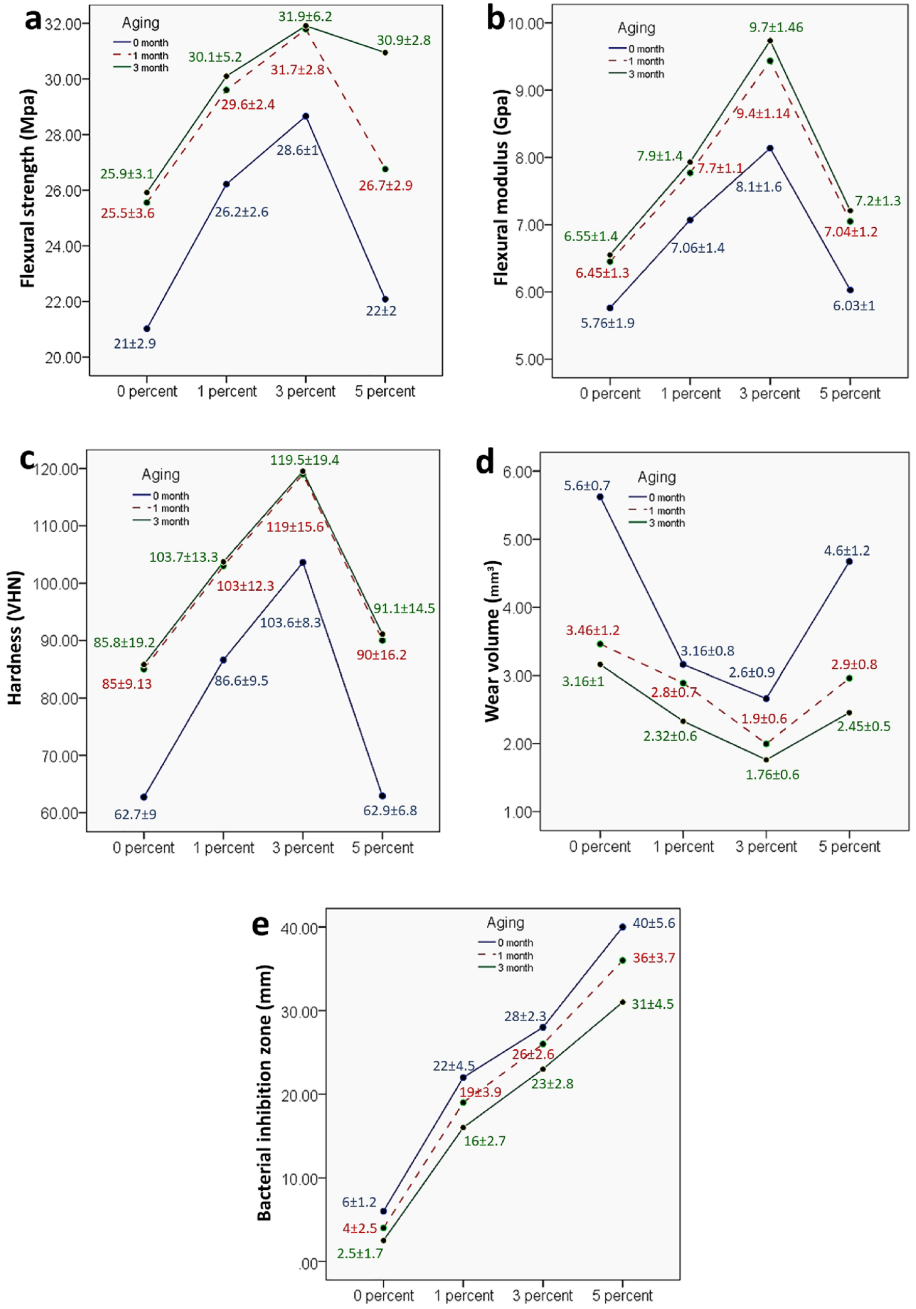
Comparison of four groups: Control group (0 percent), 1 percent group, 3 percent group and 5 percent group with respect to **a** flexural strength (MPa), **b** flexural modulus (GPa), **c** microhardness (HVN), **d** wear volume (mm^3^) and **e** bacterial inhibition zones(mm) at 0-month, 1-month and 3-month aging

Two-way ANOVA results (Fig. 6 a, b) showed non-significant interaction between the effects of filler concentration and aging periods on the flexural strength (*P* = 0.203) and the flexural modulus (*P* = 0.979). Simple main effects analysis showed that filler concentration has a statistically significant effect on both flexural strength (*p* < 0.000) and modulus (*p* < 0.000). Simple main effects analysis showed that aging has a statistically significant effect on both flexural strength (*p* < 0.000) and modulus (*p* = 0.001). Flexural strength of group 3% HTCC@MSNs showed significant improvement over control group at all times, while group 1% showed significant improvement over control group before aging and after 1 month. For group 5% HTCC@MSNs no significant increase in flexural strength at all time storage was found compared to control group. The flexural strength (MPa) values increased after 1–3 months aging for all the groups.

With regard to flexural modulus, at all storage times, group 3% HTCC@MSNs showed significant increase in the flexural modulus compared to the control group. While group 1–5% HTCC@MSNs did not show significant increase in flexural modulus compared to control group at all time storage.

The flexural modulus values increased after 1–3 months aging for all the groups. However, the difference was insignificant except for group 3% HTCC@MSNs.

#### Vickers microhardness

Two-way ANOVA results (Fig. 6 c) showed non-significant interaction between the effects of filler concentration and aging periods on microhardness (*p* = 0.749). Simple main effects analysis showed that filler concentration has a statistically significant effect on microhardness (*p* < 0.000). Simple main effects analysis showed that aging has a statistically significant effect on microhardness (*p* < 0.000). Before aging, Vickers microhardness of group 1–3% HTCC@MSNs was significantly greater than the control group. Compared to control, 5% HTCC@MSNs did not show significant increase in microhardness values. After 1- and 3-month aging periods, VHN of group 3% HTCC@MSNs was significantly greater than the control group, where group 1–5% HTCC@MSNs showed non-significant elevated values compared to control group.

The Vickers microhardness (VHN) values increased after 1–3 months aging for all the groups.

#### Wear resistance assessment

Two-way ANOVA results (Fig. 6 d) showed a significant interaction between the effects of filler concentration and aging periods on wear volumes (*p* = 0.005), which means that the relation between wear volume and filler concentration affected by the aging period. In addition, adding filler with different ratios affected the wear volume differently at each aging period. Before aging, wear volume values of group 1–3% HTCC@MSNs was significantly lower than the control group and group 5% HTCC@MSNs. The groups responded differently to aging as after 1 month aging, group 0% and group 5% HTCC@MSNs showed considerably lower wear volume than before aging. However, group 1% and group 3% HTCC@MSNs showed small difference in wear volume.

After 3-months aging periods, wear volume values of all groups decreased with nearly similar pattern.

#### Antibacterial activities

**Fig. 7:**
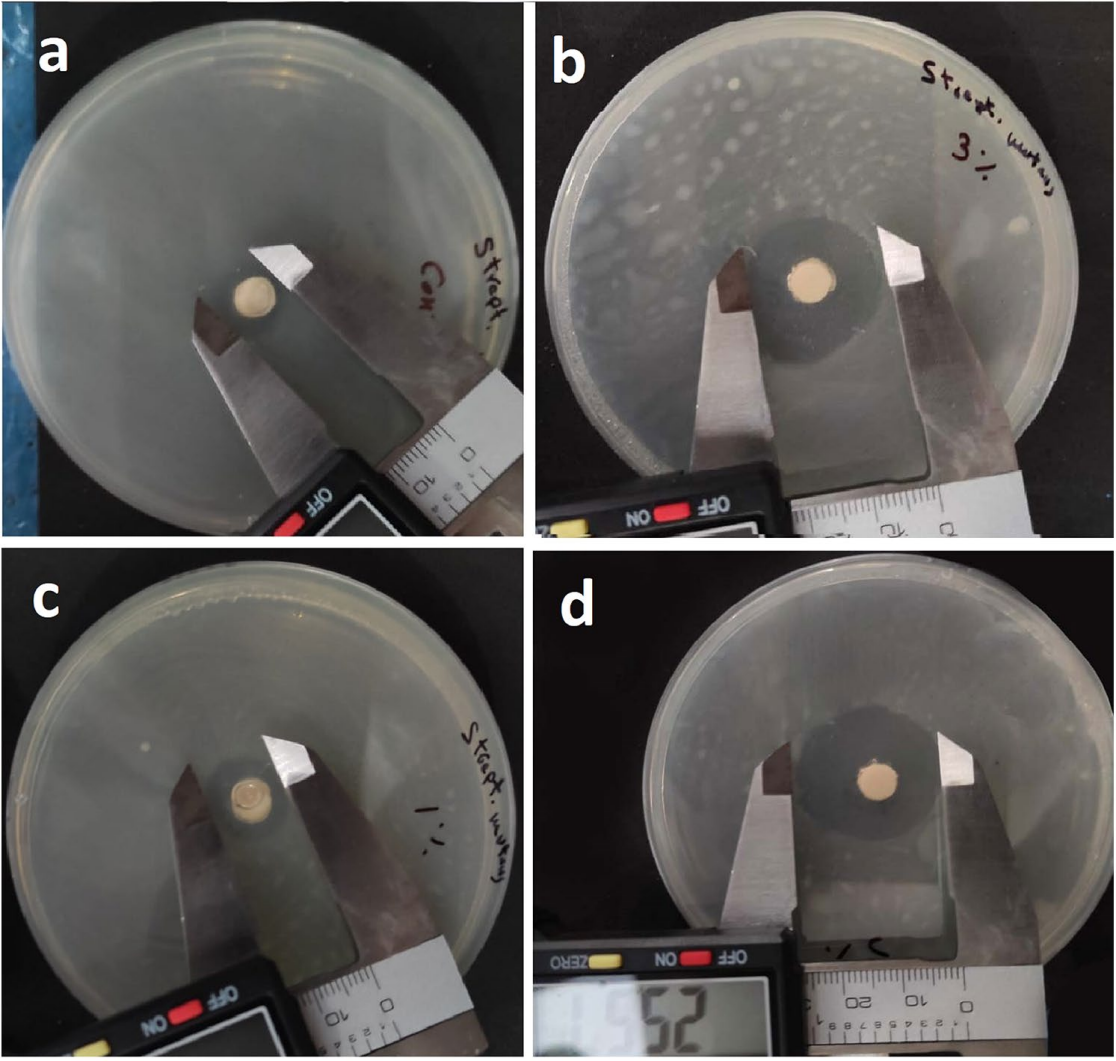
Antibacterial inhibition zones around specimens after 3-month aging

Two-way ANOVA results (Fig. 6 e) showed non-significant interaction between the effects of filler concentration and aging periods on microhardness (*p* = 0.322). Simple main effects analysis showed that filler concentration has a statistically significant effect on microhardness (*p* < 0.000). Simple main effects analysis showed that aging has a statistically significant effect on microhardness (*p* < 0.000). The bacterial inhibition zones (mm) mean value increased significantly and proportionally with the addition of HTCC@MSNs filler from 1 to 5%. Bacterial inhibition zones decreased significantly after 3-month aging for all the groups (Fig. 7).

#### Fluoride release

**Fig. 8:**
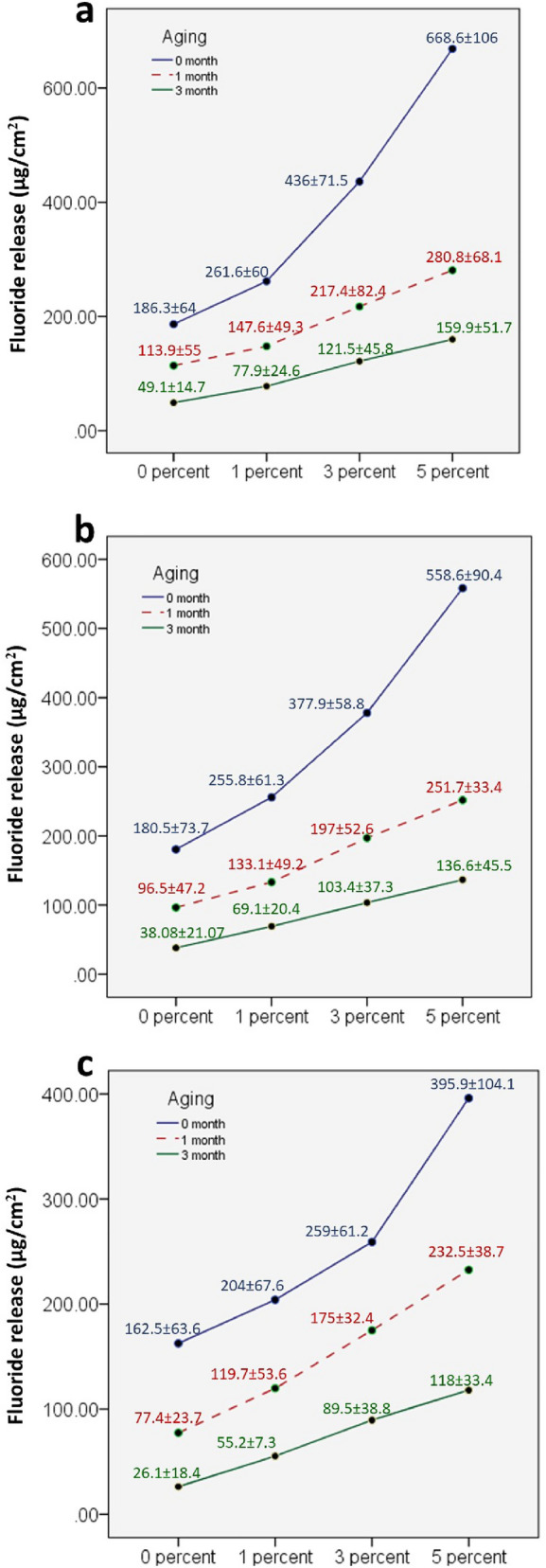
Comparison of four groups: control group (0 percent), 1 percent group, 3 percent group and 5 percent group with respect to fluoride release (μg/cm^2^) at **a** 24 h, **b** 7th day, **c** 14th day at 0-month, 1-month and 3-month aging

Two-way ANOVA results (Fig. 8) showed non-significant interaction between the effects of filler concentration and aging periods on fluoride release at 24 h, 7th day and 14th day (*p* < 0.000). Simple main effects analysis showed that filler concentration has a statistically significant effect on fluoride release at 24 h, 7th day and 14th day (*p* < 0.000). Simple main effects analysis showed that aging has a statistically significant effect on fluoride release at 24 h, 7th day and 14th day (*p* < 0.000). After 24 h, all groups showed the maximum fluoride release (μg/cm^2^) mean value, which subsequently steadily decreased until two weeks. Group 5% HTCC@MSNs showed the highest fluoride release at each time of measurement. The fluoride release (μg/cm^2^) values after 24 h, 1–2 weeks decreased after 1–3 months aging for all the groups.

## Discussion

Tooth colored restorations in posterior teeth with minimal cost are a common patient request. Glass-ionomer, because of its capability to preserve existing tooth structure and release fluoride, as well as its chemical bond to tooth structure and bulk placement, is a good candidate for improvement to attain the expected properties for patients and dentists. This study was designed to assess the hypothesis that adding HTCC@MSNs to GIC and aging would have a strengthening effect on its physico-mechanical properties and enhance its antibacterial capability and fluoride release ability.

In this study, MSNs were successfully prepared and functionalized with HTCC. Sol–gel technique was utilized due to its ability to modify the structural features (size, porosity and shape) during synthesis. The HTCC was prepared in a homogenous system to avoid a low degree of substitution with a heterogeneous reaction [24]. To enhance the degree of substitution during chitosan and GTMAC reaction, GTMAC was added in three aliquots to allow for pre-reaction. [24]. The main driving mechanism for MSN functionalization was the electrostatic attraction of positively charged HTCC with negatively charged MSNs, avoiding complex chemical grafting [23].

Flexural strength is a frequently examined mechanical property because it is more sensitive to subtle changes in the substructure than compressive strength tests. Furthermore, it allows the actual loading situation to be replicated by providing an exact assessment of a material's tensile strength. Based on the results of flexural strength tests, the HTCC@MSNs GIC performed well under flexure stress. The 3% HTCC@MSNs group demonstrated the best performance with a 36.3% increase in flexure strength compared to the control GICs. The flexural strength of experimental GIC may have been increased due: MSNs, as nanoparticles, act as a space filler, occupying the gaps between the larger glass particles. In addition, the large specific surface area of MSN allowed mechanical interlocking with the matrix, which may have resulted in stress transmission from the matrix to the MSNs. Our findings corroborated prior research on the efficacy of MSNs as fillers [7–9]. However, the flexural strength of group 5% HTCC@MSNs was lower than that of group 3% HTCC@MSNs and group 1% HTCC@MSNs, which may be attributed to that large aggregates of nanoparticles generate weak spots in the cement matrix [34].

The elastic modulus is largely concerned with choosing the right material for a certain clinical condition. Stiffer materials are best suited to the scope of occlusal repair, where material resistance must be increased. The elastic modulus increased significantly with 3 wt. % filler content. These results can be attributed to the small size of nanoparticles, the enhanced particle packing and reduced gaps and microcracks within the set cement matrix. However, increasing the filler concentration to 5 wt. % resulted in a non-significant increase in elastic modulus, which may have arised from inadequate polyacrylic ionomer to effectively engage HTCC@MSNs, which negatively impacts the interfacial adhesion between the particles and the matrix [35].

The chemical composition and microstructure of the glass, the nature, concentration, and molecular weight of the polycarboxylic acid, and the powder to liquid ratio all have a significant impact on the early strength of glass ionomer cements. After the initial setting of chemically set GICs, such as Fuji IX, the setting reaction continues for a few days in the maturation process. During this time, aluminum salt bridges form, which influences the final mechanical properties of the cements [36].

In our study, the flexural strength and flexural modulus of the control group and the experimental groups increased after aging for up to 3 months. The mechanisms that demonstrate the aging behavior of the glass ionomer cements are complex. The reinforcement probably results from formation of aluminum salt bridges, slow additional cross-linking and accumulation of the silica gel phase which results from the well-known acid degradation of the aluminosilicate glass [36].

With respect to the experimental groups, it is likely that the degree of cross-linking between polyacrylic acid and incorporated MSNs particles was also increased leading to an increase in the flexural strength and modulus. These findings are in agreement with previous studies evaluating the effect of aging on conventional types of GICs [37, 38].

The present study revealed a significant improvement in the surface hardness of GIC containing 1% and 3 wt. % HTCC@MSNs when compared to the control group, which could be further proof for MSNs strengthening impact. Hardness was shown to be higher in denser surface patterns with fewer and smaller voids. However, the lower hardness value of 5% HTCC@MSNs could be attributable to voids generated by the aggregates, which act as weak spots in the cement matrix. Due to the weakening of the bulk, these areas result in decreased hardness numbers.

Regarding hardness values after aging, the values increased up to 3-month aging for all groups. Similar results were obtained in a previous study by Zoergiebel et al. [38] that showed an increase in the hardness values after 1 month. This also was reported in another study [39]. Feng J. et al. investigated the effect of water aging for up to 6 months on the mechanical properties of modified GIC, the control group that was Fuji IX showed increase in VHN up to 3 months and then decreased at 6 months [25].

Results from the present study showed that wear-resisting performance has great improvement for HTCC@MSNs-added GIC especially the HTCC@MSNs content up to 3wt%. The results of our study agreed with a previous conclusion that wear resistance is related to mechanical properties such as elastic modulus and hardness of the materials [40].

The antimicrobial inhibition zone differed significantly between all the experimental groups and the control group. The current study found that GIC-containing HTCC@MSNs effectively inhibited bacterial growth. The size of the inhibition zones produced was correlated with the percentage of HTCC@MSNs incorporated into the GIC, with the 5% HTCC@MSNs group having the highest value. The improved antibacterial effect of HTCC@MSNs groups may be due to the ability of HTCC to diffuse and destroy the microbe with its positive charge. Areas of bacterial inhibition significantly decreased after 3 months for all groups. However, the values for the modified HTCC@MSNs groups after 3 months are high. We assume that the biofilm inhibiting properties are a combined effect from antibacterial material released and surface contact killing effect on the bacteria [25]. It was hypothesized in a previous study that the direct contact killing effect on bacteria could be the main reason for the early-stage biofilm inhibition [41]. The decrease in areas of inhibition was found in a previous study on Fuji IX after 7 days aging [42].

Fluoride release by GIC is hypothesized to be regulated by two mechanisms: a fairly quick surface burst, backed by a long-term and gradual release from the core of the material by diffusion throughout cement pores and cracks. The initial burst is desirable because of its established effect on caries prevention, whereas sustained release enhances tooth ability to resist new caries [43]. The findings of the present study are consistent with a number of in vitro investigations that found elevated fluoride release in the first two days as well [44]. However, the control group showed gradual decrease after 24 h although specimens were prepared by a standardized method. Such behavior was reported in previous studies [45, 46]. Studies using chitosan to reinforce GICs revealed that F release from GIC is catalyzed [47]. This is consistent with our recent study, as group 5% HTCC@MSNs loaded with GIC had maximum fluoride release during all time intervals, which is a beneficial effect of the new filler as this can help to inhibit dentine demineralization by a measurable amount. The findings revealed that fluoride release during the subsequent days decreased, which is consistent with other studies [44, 48]. In the present study, the fluoride release decreased significantly with age, which is related to the slower dissolution of glass particles through the pores of the material with time. This is compatible with a previous study [49]. Similar findings were found in a study of resin-modified GIC after 28 days [44].

Our study hypothesis was partially accepted; as both studied variables had a strengthening effect on its physico-mechanical properties, while their effect varied with the antibacterial capability and fluoride release ability; with the filler variable enhancing these properties and aging variable decreasing them.

Overall, we can assume that mechanical strengthening requires the filler particles to be strongly adhered to the GIC matrix in order for stress distribution to occur. Unfortunately, increasing the percentage of filler to 5wt. % may not have achieved this. The reduction in strength of group 5 percent HTCC@MSNs can be attributed to the smaller particle size and larger surface area of MSNs nanoparticles compared to glass, which may have resulted in insufficient polyacrylic ionomer to effectively bond to the increased amount of HTCC@MSNs nanoparticles powders, weakening the interfacial bonding between the particles and the ionomer matrix. However, increasing the filler content may not have hampered the filler's antibacterial or fluoride release action because neither the quaternized chitosan nor the fluoride participate in the reaction.

From the clinical point of view, although the HTCC@MSNs-modified GIC showed 36.3% increase in flexure strength compared to conventional GIC, it is still less than the required value according to ISO 4049 for polymer-based filling and restorative materials. In addition, the dynamic condition of oral cavity is expected to result in different behavior of the HTCC@MSNs-modified GIC such as: increased fluoride release in acidic attacks in the oral cavity and decreased wear volume with more effective saliva lubrication than distilled water.

There are some limitations to this study. The use of distilled water as a media for measuring fluoride release and as a lubricant for wear testing, although was used for standardization, but does not mimic the clinical situation. Although no changes in the setting time were observed during specimen preparation, its evaluation is critical because it is a practical requirement. The viscosity of the cement was not evaluated as well.

Future research should look into the physicochemical surface interactions between nanoparticles and cements to determine the optimal filler concentration needed to achieve the best properties. The mechanical and antibacterial properties of HTCC@MSNs-modified GIC should be more elucidated for longer aging periods in the future studies. Future research should focus on the fluoride recharge ability of HTCC@MSNs-modified GIC.

## Conclusion

HTCC@MSNs filler has been found to be a promising filler with strengthening and antibacterial effect which can be useful contribution to the modification of dental materials. Addition of 3 wt. % HTCC@MSNs into GIC was effective in inhibition of *Streptococcus mutans,* increasing fluoride release and improving physico-mechanical properties. Artificial aging showed a positive effect on the mechanical properties, which is believed to be due to the special setting mechanism of GICs.
